# The Importance of Heterogeneity to the Epidemiology of Tuberculosis

**DOI:** 10.1093/cid/ciy938

**Published:** 2018-11-01

**Authors:** James M Trauer, Peter J Dodd, M Gabriela M Gomes, Gabriela B Gomez, Rein M G J Houben, Emma S McBryde, Yayehirad A Melsew, Nicolas A Menzies, Nimalan Arinaminpathy, Sourya Shrestha, David W Dowdy

**Affiliations:** 1 School of Public Health and Preventive Medicine, Monash University, Melbourne, Victoria, Australia; 2 Health Economic and Decision Science, University of Sheffield, United Kingdom; 3 Liverpool School of Tropical Medicine, United Kingdom; 4 CIBIO-InBIO, Centro de Investigação em Biodiversidade e Recursos Genéticos, Universidade do Porto, Portugal; 5 Department of Global Health and Development, London School of Hygiene and Tropical Medicine, United Kingdom; 6 Tuberculosis Centre, London School of Hygiene and Tropical Medicine, United Kingdom; 7 Infectious Disease Epidemiology Department, London School of Hygiene and Tropical Medicine, United Kingdom; 8 Australian Institute of Tropical Health and Medicine, James Cook University, Townsville, Queensland; 9 Department of Global Health and Population, Harvard T. H. Chan School of Public Health, Boston, Massachusetts; 10 Department of Infectious Disease Epidemiology, School of Public Health, Imperial College London, United Kingdom; 11 Department of Epidemiology, Johns Hopkins Bloomberg School of Public Health, Baltimore, Maryland

**Keywords:** tuberculosis, heterogeneity, epidemiology, case detection, interventions

## Abstract

Although less well-recognized than for other infectious diseases, heterogeneity is a defining feature of tuberculosis (TB) epidemiology. To advance toward TB elimination, this heterogeneity must be better understood and addressed. Drivers of heterogeneity in TB epidemiology act at the level of the infectious host, organism, susceptible host, environment, and distal determinants. These effects may be amplified by social mixing patterns, while the variable latent period between infection and disease may mask heterogeneity in transmission. Reliance on notified cases may lead to misidentification of the most affected groups, as case detection is often poorest where prevalence is highest. Assuming that average rates apply across diverse groups and ignoring the effects of cohort selection may result in misunderstanding of the epidemic and the anticipated effects of control measures. Given this substantial heterogeneity, interventions targeting high-risk groups based on location, social determinants, or comorbidities could improve efficiency, but raise ethical and equity considerations.

## Teaser

Heterogeneity in tuberculosis burden is driven by the organism, host, environment, and distal determinants. More reliable data are needed given inconsistent case ascertainment. Targeting high-risk groups is an important consideration in designing interventions, but raises equity and efficiency issues.

Although estimates of the global burden of tuberculosis (TB) suggest gradual decline, this aggregate profile masks a patchy, heterogeneous epidemic that predominantly afflicts society’s most marginalized groups. Meanwhile, the causative organism is now the world’s leading infectious killer and dramatic reductions in burden will be necessary if the bold new End TB targets are to be realised [1]. Heterogeneity in disease distribution increases as the burden of an infectious disease declines and becomes more unevenly distributed across space or social networks [2]—a phenomenon that is well recognized in the case of diseases such as malaria [3]. There are many reasons to suspect that TB epidemics are highly heterogeneous, such as the prominence of highly localized or household transmission, the wide geographical variation in disease burden within and between countries, and the many individual-level factors strongly associated with risk of disease. Here we describe key drivers of heterogeneity in TB burden, discuss the challenges in quantifying this heterogeneity, and consider implications for transmission dynamics and the design of interventions.

## DRIVERS OF HETEROGENEITY

**Figure 1.**
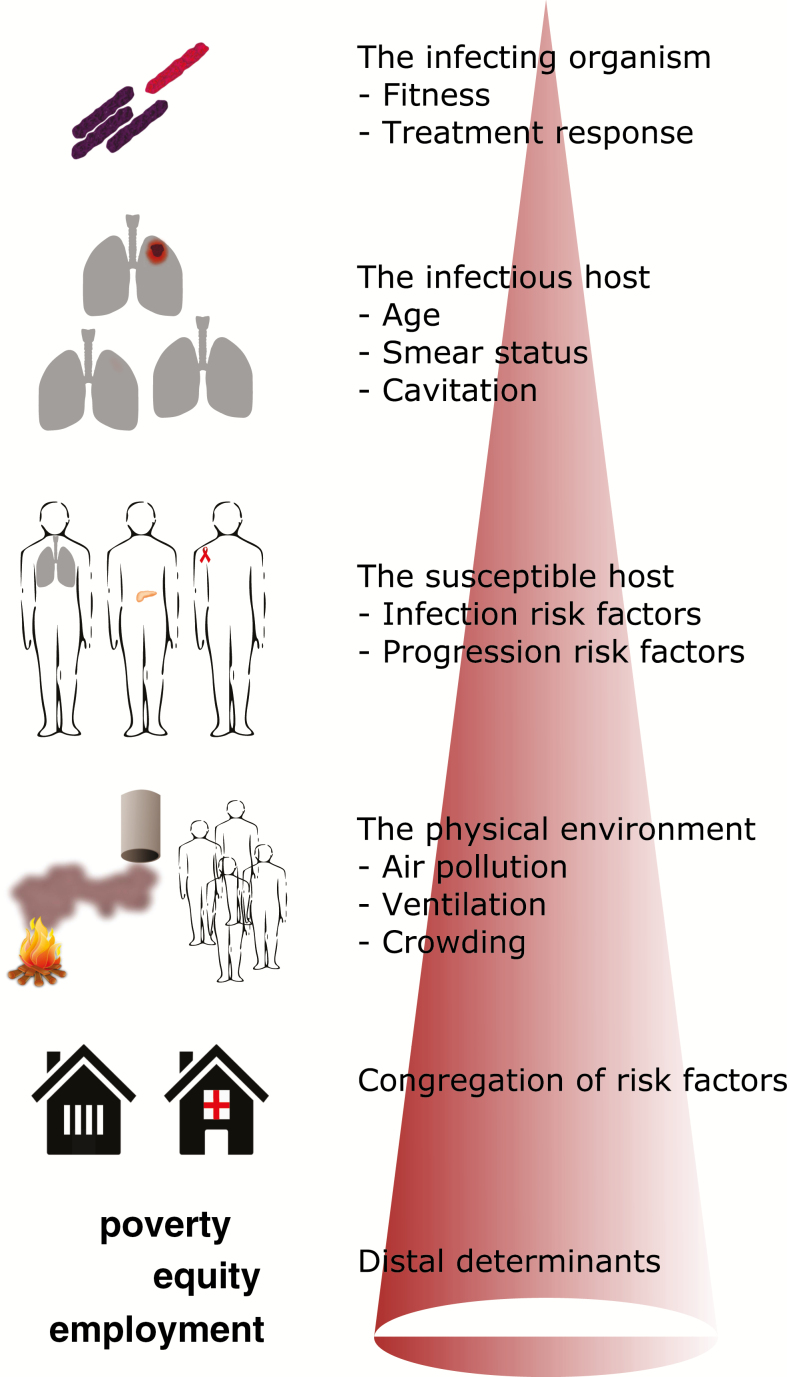
Conceptual framework for understanding heterogeneity in tuberculosis epidemiology. The cone indicates that the most local drivers are positioned toward the top of the figure and the broadest drivers toward the bottom, rather than reflecting the importance of these factors.

Risk of infectious disease is dependent on characteristics of the infectious host, the organism, the susceptible host, and the environment (Figure 1; Table 1). The complex interplay between the pathogen and the host’s immune system and the propensity for *Mycobacterium tuberculosis* (*Mtb*) to enter a latent state following infection mean that many exposed individuals will never progress to active TB disease. Therefore, individual characteristics that predispose to susceptibility to infection, progression to disease after infection, and infectiousness during disease episodes all contribute to heterogeneity, although the risk factors associated with each differ considerably. For example, risk of exposure is driven by sociodemographic factors (eg, crowding, contact patterns), susceptibility to infection once exposed is influenced by processes that impair local immune responses (eg, smoking), progression to disease may reflect systemic immune status (eg, human immunodeficiency virus [HIV], nutrition), and likelihood of onward transmission may be altered by cough symptomatology and disease duration (eg, through access to care).

**Table 1.** Examples of Specific Forms of Heterogeneity and Ways Forward

Source of Heterogeneity	Examples of Existing Evidence	Data Needs	Analytic Needs	Intervention Needs
Infectious host	Sequencing and social network analysis suggest that some individuals may act as “superspreaders” [4]	Importance of biological variables, eg, aerosolization, cough frequency	Implications of hosts with differential infectiousness and superspreading	Tools to identify the most infectious patients
	Available data on contact patterns (principally from low-burden settings) suggest age-specific (assortative) mixing	Data on contact patterns from high-burden settings and for risk factors relevant to TB (eg, HIV status)	Importance of population groups to sustaining transmission relative to their burden of disease	Case-finding efforts designed to identify patients with high-risk mixing patterns for broader dissemination of infection
Infecting organism	Strain responsible for extensive community spread confirmed to be highly virulent in mouse model [5]	Mechanisms of strain diversity and virulence	Implications of selecting for strains of greater fitness	Interventions to limit infectiousness of difficult-to-treat strains
	Highly resistant forms of TB causing extensive outbreaks, eg, XDR-TB in Tugela Ferry, South Africa [6]	Fitness costs associated with drug resistance	Likely future trajectory of drug resistance	Improved identification and treatment of highly transmissible strains of drug-resistant TB
Susceptible host	Individuals previously treated for TB had higher rates of recurrent TB due to reinfection than the general population in Cape Town, South Africa [7]	Protection or susceptibility afforded by past TB episodes and whether this is attributable to infection or progression risk	Distinguish the individual-level effect of increased susceptibility post–disease episode from the effect of selecting for a more susceptible cohort through infection	Protection of highest-risk individuals from infection or progression to disease
	Specific risk groups may experience polyclonal outbreaks [8]	Better estimates of disease prevalence in risk groups	Anticipated effects of trends in comorbid risk factors on TB	TB control interventions that link with systems for other high-risk conditions
Physical environment	Incarceration may have been a significant driver of community transmission [9]	Better estimates of location-specific TB transmission risk	Valid models for translating environmental heterogeneity into transmission risk	Active case finding targeted at high-risk environments (eg, prisons, transit)
	Greater proportion of infected contacts in less well-ventilated hospital wards [10]	Ability of specific interventions (eg, improved ventilation) to reduce that risk	Projected population-level impact of targeted environmental interventions	Mitigation of TB transmission through modification of high-risk built environments
Distal determinants	Ecological observation of declining TB rates during times of improvements in living standards [11]	Mechanistic linkages between poverty alleviation and TB transmission	Projected ability of social protection and similar efforts to reduce heterogeneity	Linkage between TB control programs and schemes to alleviate poverty and/or address other distal determinants
	Association between coverage of Brazil’s conditional cash transfer program and improved TB control [12]	TB-specific effects of broader interventions	Models of the impact of TB on other outcomes in vulnerable populations	Implementation of TB interventions in a fashion that mitigates burden on the highest-risk populations, thus promoting equity and reducing disparities in risk

Abbreviations: HIV, human immunodeficiency virus; TB, tuberculosis; XDR-TB, extensively drug-resistant tuberculosis.

### The Infectious Host

Medical and demographic factors also strongly influence the extent to which each affected person propagates *Mtb* infection. Smear-positive adults and particularly those with cavitary pulmonary TB transmit infection more extensively [13], while many others, such as those with only extrapulmonary involvement, may infect no one. Although children and persons with HIV are less likely to transmit, the degree of infectiousness is variable, with children aged >10 years more often manifesting adult forms of TB [14, 15]. Despite its limitations, smear microscopy remains the mainstay of TB diagnosis worldwide with advantages that include its ability to identify highly infectious individuals. Social factors such as mixing patterns also influence spread by modifying the number of contacts exposed and these patterns also differ by setting (eg, household, workplace, general community). Importantly, social mixing patterns may act differently for *Mtb* than for other infections, given that *Mtb*, unlike many other major pathogens, is airborne and so can be transmitted without the need for direct person-to-person contact. However, the rate of transmission per day infectious is considerably lower than for other respiratory pathogens (eg, measles, influenza) [16], meaning that amplifying factors such as cough characteristics, ability to generate aerosols of appropriate size [17], and environmental factors may strongly influence whether infection occurs. Finally, myriad programmatic and social factors delay diagnosis and so prolong the infectious period and increase the duration of exposure [18], thereby potentiating heterogeneity through their impact on the most marginalized groups.

### The Infecting Organism

*Mtb* is a clonal pathogen that displays variable fitness and a complex interaction with its human host [19]. Its multiple lineages differ in their genomic makeup and in several aspects of their clinical and epidemiological behavior, including disease progression, disease severity, transmissibility, and geographic distribution (Supplementary Bibliography). With recent advances in molecular epidemiology, the influence of *Mtb* genetic diversity on the outcomes of TB infection and disease is increasingly recognized. Strains are thought to have adapted to the human population they affect [20], resulting in a sympatric relationship whereby co-evolved host populations show high rates of TB due to certain strains, but concentration within high-risk groups elsewhere [21]. However, the discordance in findings between settings and the complex interaction between pathogen, host, and environment remain challenges to understanding these processes.

Arguably, the most critical form of pathogen-related heterogeneity is drug resistance, which makes clinical management considerably more challenging and expensive. Epidemiologically, transmission cycles of drug-resistant TB (DR-TB) differ from those of drug-susceptible TB because of limited access to the diagnostics available for determining drug resistance, the long duration of DR-TB treatment, and clustering of DR-TB patients in high-risk settings. All of these factors may act to prolong the infectious period, sustaining transmission chains of DR-TB [22]. Resistance-conferring mutations may be offset by associated physiological impairments in the organism that limit its ability to survive and reproduce (“fitness costs”), although sustained drug exposure may select for bacteria with compensatory mutations [23]. Moreover, fitness costs are likely to vary according to the drug in question (eg, higher for rifampicin resistance than for isoniazid or streptomycin) [24], while both modeling studies and large-scale outbreaks highlight the potential for DR-TB to proliferate [25].

### The Susceptible Host

Characteristics of the susceptible host also markedly influence the likelihood of disease following exposure, which may reflect both susceptibility to infection or greater risk of progression to disease for those infected. Patterns of reactivation differ markedly by age, and comorbidities such as HIV, diabetes, malnutrition, and heavy alcohol use are critical considerations in the variation of risk of disease progression observed (Supplementary Bibliography). For example, HIV is the strongest individual-level risk factor and a major driver of the TB epidemic in many parts of Africa, while the rising global prevalence of noncommunicable diseases (eg, diabetes) may hinder our ability to achieve control targets by impairing host immunity at the population level [26]. History of exposure and disease are also important, as people who are latently infected may have partial protection against reinfection with the pathogen [27], whereas previously treated persons are likely to be at substantially increased risk for recurrent disease [28]. This latter increase in risk may reflect repeated exposure, incomplete treatment, or underlying immunological vulnerability [29].

### The Physical Environment

The setting in which TB is transmitted is also an important modifier of spread—either due to increased population density or congregation of individuals with higher rates of specific risk factors, or directly through environmental features that facilitate airborne transmission. Characteristics of the physical environment that may contribute to transmission include crowding, poor ventilation, and high levels of indoor air pollution [30]. Furthermore, locations with these characteristics (eg, clinics, public transit, churches, prisons, mines, and informal drinking spaces) are often frequented by the same high-risk individuals, further fueling heterogeneous transmission in these sites. These locations are themselves likely to be in close proximity, enhancing transmission in impoverished areas [31] and sustaining the epidemic [32].

### Structural and Social Determinants

Heterogeneity at the community level is driven by a complex network of proximal and distal determinants that may not always be fully explained by quantifiable risk factors. Migration, urbanization, demographic transition, and other broad global trends combined with weak and inequitable policy and planning lead to pockets of poverty, unhealthy behaviors, and weak health systems in which TB thrives [33]. Social or spatial clustering of the individual-level characteristics described in the preceding sections may magnify the effect of these risk factors through transmission, as persons contact one another more if they share similar characteristics (assortative mixing). However, understanding of the effect of the various upstream determinants responsible for driving heterogeneity in TB burden is limited by the relative paucity of modeling studies in this area [34].

## CHALLENGES IN QUANTIFYING HETEROGENEITY

**Figure 2.**
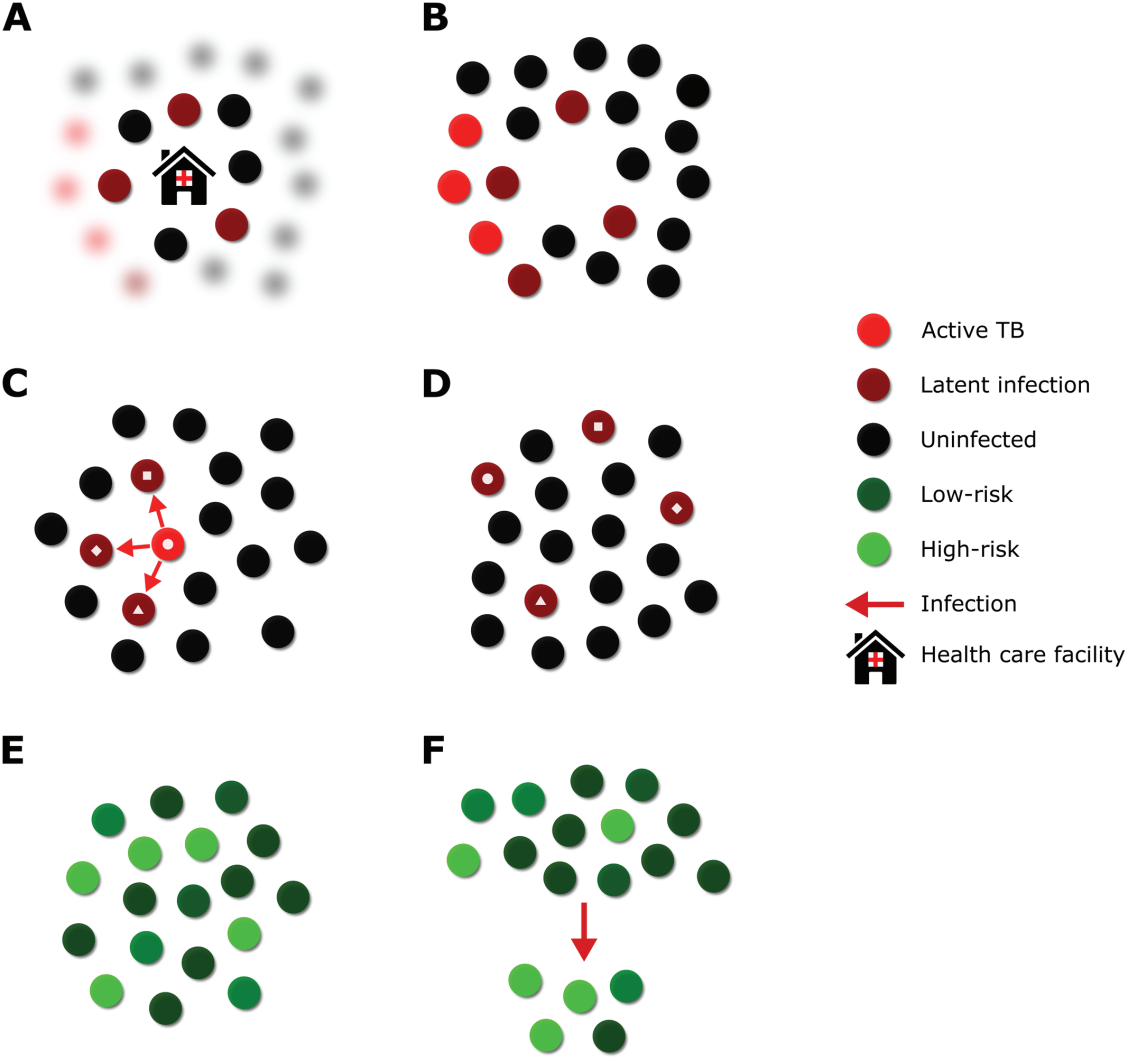
Illustration of some selected concepts from the text. *A*, Degree of heterogeneity that might be observed among individuals with good access to the healthcare system (unblurred discs) compared to those with poor access (blurred discs). This may be substantially less than the heterogeneity that exists in the population as a whole (*B*). *C*, Series of transmission events. *D*, Subsequent relocation of infected and uninfected individuals. This results in a more homogeneous distribution of infection across the population at this later time point, even though transmission was highly heterogeneous. *E*, Series of individuals at variable risk of infection. *F*, Selection of higher-risk individuals through the infection process. Although infection is the selecting illustrated process here, similar principles would apply to progression from infection to disease, through stages of the disease process and to interaction with the health system. Abbreviation: TB, tuberculosis.

Although substantial between- and within-country differences in burden are frequently reported, challenges exist in interpreting the differences observed between demographic, geographical, or other subdivisions of the population. Our understanding of the population-level epidemiology of TB disease relies to a large extent on cases that have sought care, received a diagnosis, and been recorded through surveillance systems or local studies. The substantial proportion of cases that does not reach this stage in many settings [1] means that our estimates of heterogeneity in burden are prone to bias (Figure 2A and 2B). A particular consequence of relying on data from detected cases arises from the negative correlation between TB burden and access to care, which may mask heterogeneity in disease. For example, TB prevalence surveys consistently show a male predominance among adult TB cases, but this gender gap is much smaller in notifications—suggesting that men experience a higher burden but seek or access care at a lower rate than women [35]. Similar and even stronger unobserved effects, whereby mechanisms that increase risk of TB also decrease the probability of detection, may exist for features such as socioeconomic status or locality. Moreover, even if bias could be eliminated from health information systems, routinely collected data are not typically disaggregated beyond broad age categories, geographic regions, and drug resistance profiles, thereby limiting our ability to observe heterogeneity between smaller subpopulations without specifically designed studies.

Much less biased measures of disease burden are available from the recent increase in TB prevalence surveys. However, prevalence surveys in the general population are expensive undertakings and typically designed to yield a relative precision of 20%–25% [36], limiting their ability to discern patterns among subgroups or at the district/local level. Moreover, prevalence surveys are by design cross-sectional, meaning that they cannot provide information on heterogeneity through time without additional assumptions or repeated data collection.

One important consequence of detection bias is that clusters of notifications are difficult to interpret. Apparent hotspots of TB disease may represent either true areas of intense transmission or better diagnosis (via targeted campaigns or differential access to care), such that the areas of most intense transmission may be those with the highest notification rates in some settings and the lowest in others. Travel to access care may further exaggerate this process, creating artefactual aggregations of notifications. By contrast, heterogeneity in transmission may be masked by the often substantial latent period between infection and disease onset, during which infected individuals may relocate (Figure 2C and 2D). This process smooths disease distribution and obscures transmission chains, while the distribution of transmission and latent infection are even harder to observe in an era when population-wide surveys of infection are no longer undertaken.

## IMPLICATIONS FOR UNDERSTANDING AND MODELING TRANSMISSION

The impact of heterogeneity of infectiousness is influenced by characteristics of the infectious host and the organism being transmitted, and can be explored through its specific effects on the basic reproduction number, *R*_0_ [37]. While the point estimate of *R*_0_ is often emphasized as a measure of the expected number of secondary cases caused by an average index case in an infection-naive population, infectiousness may more appropriately be viewed as a probability distribution across a population of individuals, each with their own expected number of secondary cases. While superspreading is clearly observable in TB genomic studies [38], saturation of close contacts, whereby contacts occur primarily among individuals who have already been infected, may increase the importance of community transmission in high-burden settings [39].

When heterogeneity in susceptibility to TB exists, concerns regarding the assumption of a homogeneous population parallel concepts familiar in noncommunicable diseases, such as cohort selection and frailty models in survival analysis. As higher-risk individuals develop incident disease [28, 40], the incidence rate of a cohort may decline simply because those who remain susceptible have a lower average risk (Figure 2E and 2F). This process is disabled in models that collapse risk distributions to their mean values, leading to inaccurate simulations and biased predictions. Population-level heterogeneity in susceptibility can also induce thresholds near which small epidemiological changes will cause dramatic shifts in disease burden, leading to unanticipated effects of preventive interventions [41] and faster emergence of drug-resistant strains [42].

**Figure 3.**
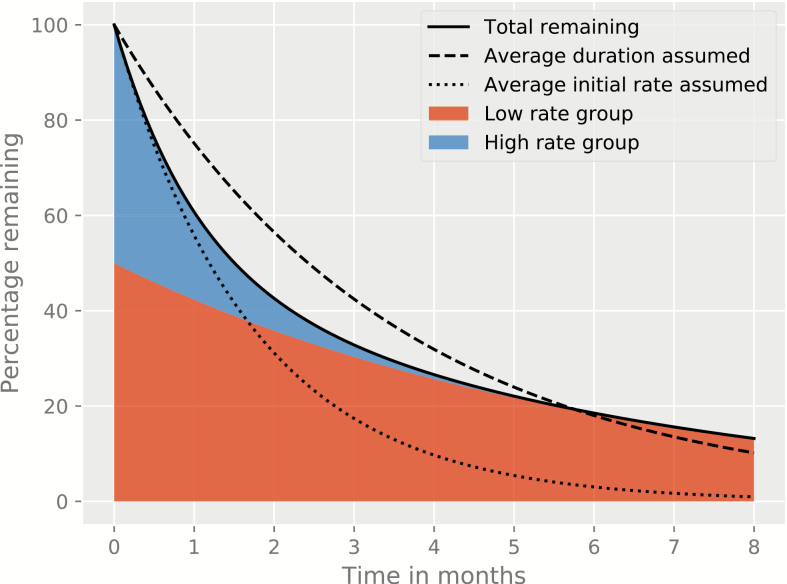
Composition of a simple 2-stratum heterogeneous cohort over time from entry to an epidemiological state (active undiagnosed tuberculosis). Plot displays the percentage of patients with active tuberculosis remaining undiagnosed after the onset of infectiousness (time 0 on the horizontal axis), under the assumption that 50% of the initial cohort has an average duration of infectiousness of 1 month (high-rate group), and 50% of the cohort has a duration of infectiousness of 6 months (low-rate group). The true total percentage of patients remaining infectious with time since onset of infectiousness (solid line) is compared against the proportion that would be expected to remain if the whole cohort was assumed to have the average time to diagnosis (3.5 months), and the proportion that would be expected to remain if the whole cohort was assumed to have a rate of diagnosis that is the average of the rates of the 2 groups (dotted line). The amount of the total population comprised of high-rate and low-rate persons at each time point is indicated by colored shading, demonstrating that the remaining cohort is increasingly comprised of low-rate individuals over time.

Any transition rate can be affected by cohort selection, as illustrated in Figure 3. Instead of the disease incidence process discussed above, consider a cohort of individuals with active TB comprised of 2 groups: fast and slow care seekers. As the faster care seekers leave the cohort earlier, the overall care-seeking rate will decline over time, even though it remains constant in each group. This process complicates estimation procedures and can be especially problematic in relation to rates of infection, which are proportional to the prevalence of infectious individuals and so part of a feedback loop. Moreover, epidemiological uncertainty around the most appropriate parameter values for transmission models means that multiple parameter sets may superficially replicate observed burden [43], which is particularly problematic for an endemic infection with a prolonged and unpredictable latency period.

## IMPLICATIONS FOR CONTROL

### Targeting Risk Groups

A consequence of the heterogeneity in transmission, infection, incidence, and mortality is that benefits of interventions will differ depending on the groups targeted and the distribution of the risk factors introduced above. This consideration motivates much current TB policy, with groups at higher risk of infection, disease, or poor outcomes from TB episodes, such as household contacts, children, persons living with HIV, individuals with end-stage renal disease, and previously treated people identified as high-priority groups for screening and treatment of latent and active TB (Supplementary Bibliography). Heterogeneity in historical TB exposure is also a focus of interventions, with many low-incidence countries targeting services to foreign-born individuals [44], given their higher prevalence of latent TB and consequent risk of reactivation. However, interventions targeted at high-risk populations have not always been successful: A trial of mass screening and preventive treatment in South African miners had no impact on TB rates [45], because of reactivation of noncured infections and reinfection in the context of insufficient treatment and ongoing high environmental transmission risk [46].

### Synergies With Non-TB Interventions

Regular interactions with the healthcare system for the management of chronic and noncommunicable diseases offer the opportunity for intensified case finding efforts, given that many such conditions increase TB risk or co-occur in populations with such increased risk. More broadly, strengthening health systems for both TB and noncommunicable disease control provides the potential for synergistic interventions across diseases [47], while improving control by addressing distal determinants should also be a high priority [48]. The observation that both historical and more recent declines [11, 33] in TB burden have usually been achieved in the context of improvements in socioeconomic indicators highlights the importance of such upstream determinants and is particularly relevant in the era of the United Nations Sustainable Development Goals.

### Geographical Targeting

TB incidence shows considerable geographical clustering at multiple resolutions [49], and spatial targeting of interventions has the potential to achieve major reductions in burden through focusing on geographically discernible TB hotspots [50]. However, the extent of mixing between hotspots and the broader population is important to quantify as it will modify the impact of such interventions [51]. Intensive TB control interventions targeted at Inuit communities in northern Canada, Alaska, and Greenland were effective at substantially reducing the extreme rates of TB incidence and mortality observed in the 1950s [52]. New and emerging analytic tools offer opportunities to identify and quantify TB hotspots, such as a recent genomic analysis in Peru that highlighted the spatial aggregation of multidrug-resistant genotypes [32].

### Effect of Interventions on Heterogeneity

Where substantial reductions in TB burden are achieved, heterogeneity in TB distribution may increase, as transmission becomes more localized to remaining regions and population groups with fewer resources, limited healthcare access, and insufficient adherence to policy. However, even when fully implemented, control efforts may increase or decrease transmission heterogeneity depending on the intervention design. Interventions directed at those with poor access to care and thus high burden of disease may reduce heterogeneity, whereas interventions that strengthen routine programmatic management may increase heterogeneity even while decreasing overall burden. Heterogeneity may modify the impact of both targeted and untargeted interventions depending on the background burden of disease. For example, successful detection and treatment of a single active case may eliminate transmission from a community in a low-burden setting, whereas this would be harder to achieve in a high-burden setting. This may lead to unexpected relationships between control efforts and consequent reduction in the annual risk of *Mtb* infection [53].

### Economic and Equity Concerns

The targeting of TB control interventions to those with high rates of infection or disease is expected to increase the effectiveness of interventions. Consequent gains in efficiency will depend on coverage levels, accessibility, disease prevalence, and contribution to transmission in the wider population of the target group. There are economies of scale to be achieved when increasing coverage, yet at high levels of coverage or for difficult-to-reach populations, targeted strategies may require additional supporting activities and so increase resource needs. For example, the cost-effectiveness of active case finding strategies is driven by both the heterogeneity in disease rates and in the cost of reaching different subgroups [54]. While maximizing impact within a given budget is a key objective in priority setting, heterogeneity in burden, healthcare access, and financial resources are linked to equity concerns in resource allocation for TB control strategies. Conceptually, the difference between inequalities and inequities is a value judgement about whether the observed heterogeneity is considered fair. Policy makers should seek to ensure that populations already experiencing increases in risk due to socioeconomic or other conditions (eg, crowding, incarceration) do not experience additional disparities in access to TB diagnosis and treatment, financial burden of illness, or unwarranted exposure to infection. While the reduction of such disparities is a key policy objective, there are situations in which achieving it may imply trade-offs in efficiency gains. For example, interventions aiming to place new technologies at decentralized locations may not be as cost-effective as placement at higher levels of the health system, yet may still be prioritized to reduce social inequities in financial burden, health outcomes, and access to health services [55].

## WAYS FORWARD AND CONCLUSIONS

Causes of heterogeneity in TB epidemiology are diverse and include characteristics of the infectious host, pathogen, susceptible host, environment, and distal determinants—factors that may interact to amplify or reduce heterogeneity. Observed heterogeneity may not reflect reality and targeted epidemiological studies to quantify disease burden in more detail would be valuable, for example, prevalence surveys powered to obtain precise estimates of disease burden in specific population risk groups and age groups.

All TB modeling studies must judge which aspects of heterogeneity are sufficiently important to include given the question posed and the local context, and those which should not be specifically incorporated for parsimony. This highlights the importance of (1) detailed, context-specific data; (2) refining parameter estimation through epidemiological research; (3) communicating uncertainty in predictive modeling; and (4) confirmation of the predicted effectiveness and cost of interventions through operational research.

Heterogeneity has implications for the effectiveness and efficiency of control interventions. Targeting of interventions is an appropriate consideration in designing intervention strategies, although evidence to support specific targeted approaches is sometimes weak or contradictory. Therefore, such strategies must be considered in the context of resource availability and the ethical imperative to ensure universal access to high-quality care. Moreover, it is also important to balance the need for clear guidelines that can facilitate the broad implementation of interventions at a national or global level against the importance of developing interventions that are targeted toward specific characteristics of regional or local epidemics.

As the global TB control community looks toward ending TB, understanding and harnessing heterogeneity to improve control will become increasingly important. Key considerations in addressing heterogeneity include better assessment of disease burden in population subgroups, context-specific modeling, targeting of interventions, and a focus on distal determinants of inequities in health status.

## Supplementary Data

Supplementary materials are available at *Clinical Infectious Diseases* online. Consisting of data provided by the authors to benefit the reader, the posted materials are not copyedited and are the sole responsibility of the authors, so questions or comments should be addressed to the corresponding author.

ciy938_suppl_Supplementary_Material

## Notes

***Author contributions.*** J. M. T., P. J. D., Y. A. M., and D. W. D. coordinated this project. M. G. M. G. drafted the “Implications for Transmission Dynamics section’’. G. G. drafted the “Economic and Equity Concerns section’’. R. M. G. J. H. drafted the ‘‘Physical Environment section’’. N. A. M. drafted the ‘‘Effect of Interventions on Heterogeneity section’’. N. A. drafted the ‘‘Targeting Risk Groups section’’. S. S. drafted the ‘‘Infectious Host section’’. Y. A. M. drafted the ‘‘Ways Forward and Conclusions section’’. J. M. T. wrote the first draft of the full manuscript, which all authors reviewed and edited before submission.

***Financial support.*** This perspective piece arose out of discussions of the Modelling Research Group at the annual meeting of the TB Modelling and Analysis Consortium, which is funded by the Bill & Melinda Gates Foundation. J. M. T. is a recipient of an Early Career Fellowship from the National Health and Medical Research Council, Australia (fellowship number APP1142638). R. M. G. J. H. received funding from the European Research Council under the European Union’s Horizon 2020 research and innovation programme (grant agreement number 757699). P. J. D. is supported by the Medical Research Council, United Kingdom (fellowship number MR/P022081/1).

***Potential conflicts of interest.*** All authors: No reported conflicts of interest. All authors have submitted the ICMJE Form for Disclosure of Potential Conflicts of Interest. Conflicts that the editors consider relevant to the content of the manuscript have been disclosed.
