# Supplementary material for: The Importance of Heterogeneity to the Epidemiology of Tuberculosis
Source: Clin Infect Dis. 2018 Nov 1;69(1):159–66. doi: 10.1093/cid/ciy938 (PMC6579955; doi:10.1093/cid/ciy938)
Supplement: ciy938_suppl_Supplementary_Material [file ciy938_suppl_supplementary_material.docx]

## Supplement

### Supplemental bibliography

| Pathogen genetic variation in disease progression rates | de Jong BC, Hill PC, Aiken A, et al. Progression to active tuberculosis, but not transmission, varies by Mycobacterium tuberculosis lineage in The Gambia. J Infect Dis **2008**; 198:1037–43. Available at: https://academic.oup.com/jid/article-lookup/doi/10.1086/591504. Accessed 25 March 2018. |
| --- | --- |
| Pathogen genetic variation in disease severity | Nahid P, Bliven EE, Kim EY, et al. Influence of M. tuberculosis Lineage Variability within a Clinical Trial for Pulmonary Tuberculosis. PLoS One **2010**; 5:e10753. Available at: http://dx.plos.org/10.1371/journal.pone.0010753. Accessed 25 March 2018. |
| Pathogen genetic variation in transmissibility | Cowley D, Govender D, February B, et al. Recent and Rapid Emergence of W-Beijing Strains of Mycobacterium tuberculosis in Cape Town, South Africa. Clin Infect Dis **2008**; 47:1252–1259. Available at: http://www.ncbi.nlm.nih.gov/pubmed/18834315. Accessed 25 March 2018. |
| Pathogen genetic variation in geographic distribution | Reed MB, Pichler VK, McIntosh F, et al. Major Mycobacterium tuberculosis lineages associate with patient country of origin. J Clin Microbiol **2009**; 47:1119–28. Available at: http://www.ncbi.nlm.nih.gov/pubmed/19213699. Accessed 25 March 2018. |
| Pathogen genetic variation in outcome of infection | Coscolla M, Gagneux S. Consequences of genomic diversity in Mycobacterium tuberculosis. Semin Immunol **2014**; 26:431–444. Available at: http://www.ncbi.nlm.nih.gov/pubmed/25453224. Accessed 24 March 2018. |
| Variation in reactivation profile by age | Trauer JM, Moyo N, Tay EL, et al. Risk of active tuberculosis in the five years following infection ??? 15%? Chest **2016**; 149:516–525. |
| Variation in reactivation profile by HIV status | Kwan CK, Ernst JD. HIV and tuberculosis: a deadly human syndemic. Clin Microbiol Rev **2011**; 24:351–76. Available at: http://www.ncbi.nlm.nih.gov/pubmed/21482729. Accessed 7 March 2017. |
| Variation in reactivation profile by diabetes status | Odone A, Houben RMGJ, White RG, Lönnroth K. The effect of diabetes and undernutrition trends on reaching 2035 global tuberculosis targets. Lancet Diabetes Endocrinol **2014**; 2:754–764. Available at: http://www.ncbi.nlm.nih.gov/pubmed/25194888. Accessed 4 August 2017. |
| Variation in reactivation profile by malnutrition status | Cegielski JP, McMurray DN. The relationship between malnutrition and tuberculosis: evidence from studies in humans and experimental animals. Int J Tuberc Lung Dis **2004**; 8:286–98. Available at: http://www.ncbi.nlm.nih.gov/pubmed/15139466. Accessed 26 September 2018. |
| Variation in reactivation profile by alcohol intake | Lonnroth K, Williams BG, Stadlin S, Jaramillo E, Dye C. Alcohol use as a risk factor for tuberculosis - a systematic review. BMC Public Health **2008**; 8:289. Available at: http://www.ncbi.nlm.nih.gov/pubmed/18702821. |
| Contribution of crowding to transmission | Pelissari DM, Diaz-Quijano FA. Household crowding as a potential mediator of socioeconomic determinants of tuberculosis incidence in Brazil. PLoS One **2017**; 12:e0176116. Available at: http://dx.plos.org/10.1371/journal.pone.0176116. Accessed 26 April 2018. |
| Contribution of poor ventilation to transmission | Lygizos M, Shenoi S V, Brooks RP, et al. Natural ventilation reduces high TB transmission risk in traditional homes in rural KwaZulu-Natal, South Africa. BMC Infect Dis **2013**; 13:300. Available at: http://www.ncbi.nlm.nih.gov/pubmed/23815441. Accessed 26 April 2018. |
| Contribution of indoor air pollution to transmission | Lin H-H, Ezzati M, Murray M. Tobacco Smoke, Indoor Air Pollution and Tuberculosis: A Systematic Review and Meta-Analysis. PLoS Med **2007**; 4:e20. Available at: http://dx.plos.org/10.1371/journal.pmed.0040020. Accessed 26 April 2018. |
| Intense transmission in public transportation | Andrews JR, Morrow C, Wood R. Modeling the Role of Public Transportation in Sustaining Tuberculosis Transmission in South Africa. Am J Epidemiol **2013**; 177:556–561. Available at: http://www.ncbi.nlm.nih.gov/pubmed/23423215. Accessed 30 April 2018. |
| Intense transmission in community hotspots | Murray EJ, Marais BJ, Mans G, et al. A multidisciplinary method to map potential tuberculosis transmission ‘hot spots’ in high-burden communities. Int J Tuberc Lung Dis **2009**; 13:767–74. Available at: http://www.ncbi.nlm.nih.gov/pubmed/19460255. Accessed 26 April 2018. |
| Intense transmission in prisons | Stuckler D, Basu S, McKee M, King L. Mass incarceration can explain population increases in TB and multidrug-resistant TB in European and central Asian countries. Proc Natl Acad Sci U S A **2008**; 105:13280–5. Available at: http://www.ncbi.nlm.nih.gov/pubmed/18728189. Accessed 16 January 2018. |
| Potential effectiveness of targeting household contacts | Fox GJ, Barry SE, Britton WJ, Marks GB. Contact investigation for tuberculosis: a systematic review and meta-analysis. Eur Respir J **2013**; 41:140–156. Available at: http://www.ncbi.nlm.nih.gov/pubmed/22936710. |
| Potential effectiveness of targeting children | Marais BJ, Gie RP, Schaaf HS, et al. The natural history of childhood intra-thoracic tuberculosis: a critical review of literature from the pre-chemotherapy era. Int J Tuberc Lung Dis **2004**; 8:392–402. Available at: http://www.ncbi.nlm.nih.gov/pubmed/15141729. |
| Potential effectiveness of targeting persons living with HIV | Houben RMGJ, Crampin AC, Ndhlovu R, et al. Human immunodeficiency virus associated tuberculosis more often due to recent infection than reactivation of latent infection. Int J Tuberc Lung Dis **2011**; 15:24–31. Available at: http://www.ncbi.nlm.nih.gov/pubmed/21276292. Accessed 26 March 2018. |
| Potential effectiveness of targeting persons with end-stage renal disease | Fang HC, Lee PT, Chen CL, Wu MJ, Chou KJ, Chung HM. Tuberculosis in patients with end-stage renal disease. Int J Tuberc Lung Dis **2004**; 8:92–7. Available at: http://www.ncbi.nlm.nih.gov/pubmed/14974751. Accessed 26 March 2018. |
| Potential effectiveness of targeting previously treated patients | Marx FM, Yaesoubi R, Menzies NA, et al. Tuberculosis control interventions targeted to previously treated people in a high-incidence setting: a modelling study. Lancet Glob Heal **2018**; 6:e426–e435. Available at: https://www.sciencedirect.com/science/article/pii/S2214109X18300226#fig2. Accessed 5 June 2018. |

### Code

The following is the code used to produce Figure 3, written in Python 3.6.

from numpy import linspace, mean, exp, zeros, log

import matplotlib.pyplot as pyplot

# set parameters (months is assumed to be the unit of time)

low_risk_duration = 6.

high_risk_duration = 1.

max_x_value = 8.

# calculations

low_risk_rate = 1. / low_risk_duration

high_risk_rate = 1. / high_risk_duration

x_values = linspace(0., max_x_value, 101)

low_risk_values = 50. * exp(-low_risk_rate * x_values)

high_risk_values = 50. * exp(-high_risk_rate * x_values)

average_initial_rate = mean([low_risk_rate, high_risk_rate])

average_duration_rate = 1. / ((low_risk_duration + high_risk_duration) / 2.)

average_risk_values = 100. * exp(-average_initial_rate * x_values)

average_duration_values = 100. * exp(-average_duration_rate * x_values)

total_risk = [i + j for i, j in zip(low_risk_values, high_risk_values)]

fitted_rate = log(total_risk[-1] / 100.) / -max_x_value

targeted_rate = 100. * exp(-fitted_rate * x_values)

# create data for patches

x_values_back_and_forth = list(x_values[::-1]) + list(x_values)

low_risk_patch_matrix = list(zeros(len(x_values))) + list(low_risk_values)

high_risk_patch_matrix = list(low_risk_values[::-1]) + list(total_risk)

# plot

pyplot.style.use('ggplot')

figure = pyplot.figure()

axis = figure.add_subplot(111)

axis.fill_between(x_values_back_and_forth, low_risk_patch_matrix, label='Low rate group', alpha=.8)

axis.fill_between(x_values_back_and_forth, high_risk_patch_matrix, label='High rate group', alpha=.8)

axis.plot(x_values, total_risk, color='k', label='Total remaining')

axis.plot(x_values, average_duration_values, color='k', linestyle='--', label='Average duration assumed')

axis.plot(x_values, average_risk_values, color='k', linestyle=':', label='Average initial rate assumed')

# axis.plot(x_values, targeted_rate, color='k', linestyle='-.', label='Targeted rate')

axis.set_xlabel('Time in months')

axis.set_ylabel('Percentage remaining')

axis.legend()

figure.savefig('heterogeneity_figure_3', dpi=500)

# report the rates

print('Rate to reach correct proportion at end of displayed period is %0.2f' % (1. / fitted_rate))

print('Average initial rate is %0.2f' % (1. / average_initial_rate))

print('Average duration rate is %0.2f' % (1. / average_duration_rate))
